# Intramedullary leukocytoclastic vasculitis and neutrophil extracellular trap (NET) formation in POEMS syndrome

**DOI:** 10.1007/s00277-024-05651-w

**Published:** 2024-03-04

**Authors:** Wiebke Aderhold, Benjamin Lenz, Marc P. Hübner, Hans-Eckart Schaefer, Florian C. Gaertner, Annkristin Heine, Ines Gütgemann

**Affiliations:** 1 Institute of Pathology, University Hospital Bonn, Bonn, Germany; 2 Institute for Medical Microbiology, Immunology and Parasitology, University Hospital Bonn, Bonn, Germany; 3 German Center for Infection Research (DZIF), Partner Site, Bonn-Cologne, Bonn, Germany; 4 Department of Pathology, University Hospital Freiburg, Freiburg, Germany; 5 Clinic for Nuclear Medicine, University Hospital Bonn, Bonn, Germany; 6 Medical Clinic III for Hematology, Oncology, Rheumatology and Stem Cell Transplantation, University Hospital Bonn, Bonn, Germany

POEMS syndrome [1] is a rare paraneoplastic syndrome caused by clonal plasma cells or lymphoplasmacytic cells causing polyneuropathy, organomegaly, endocrinopathy/edema, and monoclonal-paraprotein. Most clinical and histopathologic findings can be explained by massive vascular endothelial growth factor (VEGF) secretion [2]. NETosis is a program for formation of neutrophil extracellular traps (NETs), which consist of release of decondensed chromatin and granular contents extracellularly from granulocytes and monocytes. Here, we demonstrate leukocytoclastic vasculitis and NETosis in the bone marrow of an 80-year-old patient with POEMS syndrome. This report represents, to the best of our knowledge, the first published case of leukocytoclastic vasculitis and NETosis in POEMS syndrome and supports the role of VEGF in this phenomenon.

The patient presented with lower back pain, fatigue, and monoclonal gammopathy of undetermined significance; IgA lambda type (MGUS) was noted.

Polyneuropathy (dysesthesia, abnormal electroneuronography), papilledema, type II diabetes, metabolically active sclerotic bone lesions (Fig. 1a), enlarged lymph nodes, type B symptoms, but no hepatosplenomegaly or skin lesions were found clinically. Laboratory findings included elevated CRP (80 mg/l) and significantly elevated VEGF levels of > 2000 pg/ml (normal < 380 pg/ml).

**Fig. 1:**
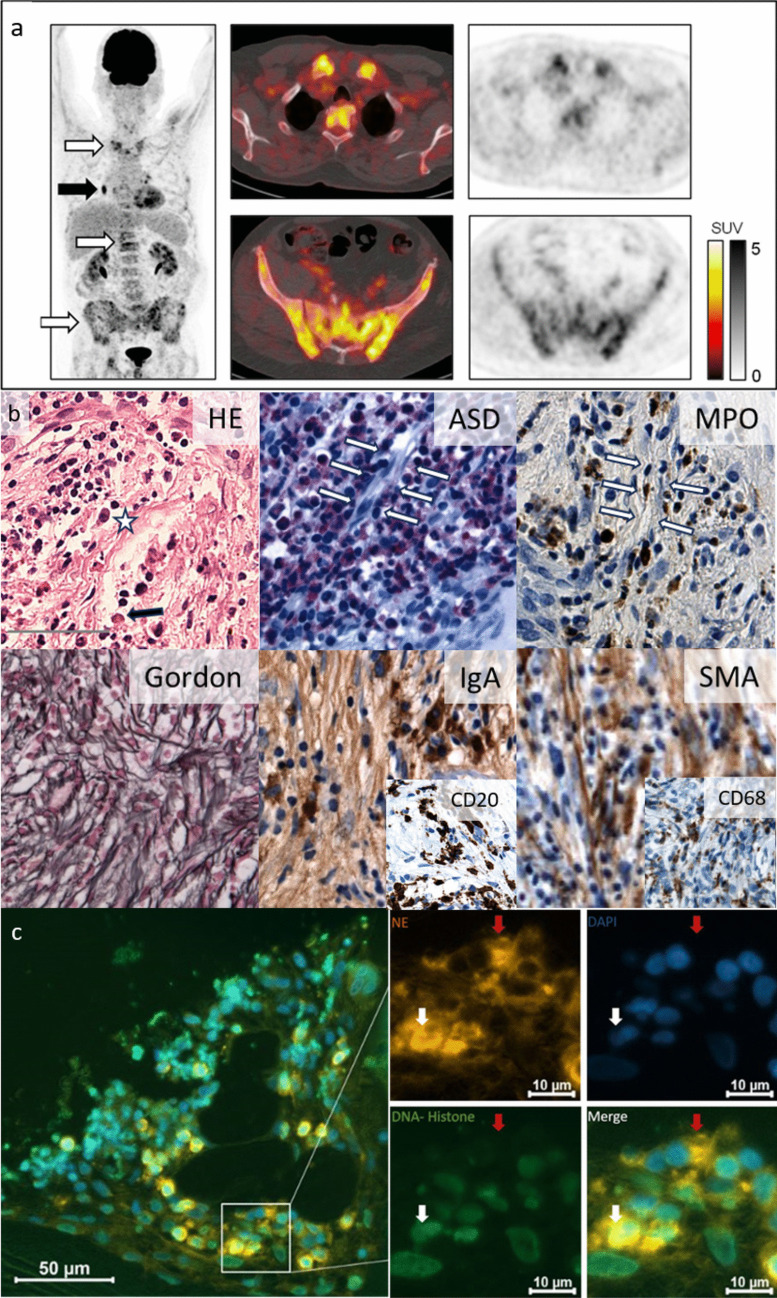
Multifocal myelofibrosis and osteosclerosis in POEMS, histology, radiology, immunohistochemistry, and NETosis by immunofluorescence. **a** FDG PET-CT showing hypermetabolic osteosclerotic pelvic, vertebral, and clavicular bone lesions (white arrows) and hypermetabolic bi-hilar lymphadenopathy (black arrow). **b** Histopathologic findings, bone marrow biopsy: leukocytoclastic vasculitis (white arrows) with prominent vessels and perivascular mature neutrophils (ASD: chloracetic esterase, MPO: myeloperoxidase) and patchy fibrosis, displacing normal hematopoiesis (HE: hematoxylin eosin); neoplastic cells consisting of CD20 positive lymphocytes and IgA positive plasma cells and lymphoplasmacytic transitions, plasma cell (star), Dutcher body (black arrow); admixed and adjacent storiform collagen-rich fibrosis (Gordon silver stain); SMA highlighting myofibroblasts, CD68 abundant macrophages in immunohistochemistry Sects. (400 × , scale bar represents 50 μm) [9]. **c** Immunofluorescence microscopy (methods see Ref. [10]). Overview of neutrophil-elastase (NE) and DNA-histone complex, DAPI (blue) (164 × axio-observer). Insert: scattered neutrophil nuclei (DNA-histone) colocalizing with NE staining demonstrating NETosis (white arrow). Red arrows: extracellular NE in degranulating neutrophils. Single channels and merged image

Iliac crest biopsy histologically (Fig. 1b) apparently captured one of the osteosclerotic foci revealed by positron emission tomography-computed tomography (PET-CT) (Fig. 1a), as peripheral blood counts were near normal apart from mildly elevated thrombocytes (400 g/l). Myofibroblast proliferation (Fig. 1b, SMA) and osteosclerosis with broadening of unmineralized osteoid activated osteoclasts, osteoblasts, and increased macrophages (Fig. 1b, CD68) were seen, reminiscent of osteomyelitis and distinct from myelofibrosis in myeloproliferative neoplasms. Osteosclerotic inflammatory lesions within ischemic medullary areas contained lymphocytes admixed with IgA + lambda restricted plasma cells (Fig. 1b, CD20, IgA).

Polyneuropathy, MGUS, sclerotic bone lesions, and elevated VEGF levels were defined as major, endocrinopathy, thrombocytosis, and lymphadenopathy as minor criteria according to the IWMG [1] for POEMS syndrome. Therapy composed of dexamethasone p.o. and bortezomib s.c. for six cycles led to a significant reduction of VEGF levels to 705 pg/ml, normalization of platelet counts and CRP, and reduced metabolic activity of the bone lesions, weight gain, and less fatigue.

Within the bone marrow leukocytoclastic vasculitis and focal NETosis was demonstrated (Fig. 1b, c). By immunofluorescence, pockets of neutrophils and distorted nuclei (DAPI) colocalizing with neutrophil elastase (NE) and DNA-histone (Fig. 1c, white arrows) supported NETosis in situ.

Leukocytoclastic vasculitis is a hypersensitivity vasculitis. It occurs as a complex series of endothelial/leukocyte interactions, vascular dilation, and leakage. Deposition of immune complexes and C3 around blood vessels is involved in the damage to endothelial cell membranes [3]. Interestingly, in patients with leukocytoclastic vasculitis in the skin, VEGF serum levels are also elevated [4]. One explanation for degranulation of mature neutrophils, NETosis, and leukocytoclastic vasculitis in POEMS may be via phosphorylation of ERK (extracellular-signal regulated kinases) downstream of VEGF receptor 1/flt1 in neutrophils [5, 6].

While in vitro NETosis is a broadly studied effect and has been demonstrated to play a pivotal role in severe COVID-19 [7], few studies have shown NETosis occurring in vivo [8]. Further studies are needed to investigate the exact mechanism of leukocytoclastic vasculitis and NETosis and whether anti-VEGF inhibition may be of therapeutic use in a subset of patients.

## Data Availability

Additional data is available upon request.
